# Signal transduction in neurons: effects of cellular prion protein on fyn kinase and ERK1/2 kinase

**DOI:** 10.1186/1742-4933-7-S1-S5

**Published:** 2010-12-16

**Authors:** Vittorio Tomasi

**Affiliations:** 1 Department of Experimental Biology, University of Bologna Via Selmi, 3 40126 – Bologna, Italy

## Abstract

**Background:**

It has been reported that cellular prion protein (PrP^c^) co-localizes with caveolin-1 and participates to signal transduction events by recruiting Fyn kinase. As PrP^c^ is a secreted protein anchored to the outer surface membrane through a glycosylphosphatidylinositol (GPI) anchor (^sec^PrP) and caveolin-1 is located in the inner leaflet of plasma membrane, there is a problem of how the two proteins can physically interact each other and transduce signals.

**Results:**

By using the GST-fusion proteins system we observed that PrP^c^ strongly interacts with caveolin-1 scaffolding domain and with a caveolin-1 hydrophilic C-terminal region, but not with the caveolin-1 N-terminal region. In vitro binding experiments were also performed to define the site(s) of PrP^c^ interacting with cav-1. The results are consistent with a participation of PrP^c^ octapeptide repeats motif in the binding to caveolin-1 scaffolding domain. The caveolar localization of PrP^c^ was ascertained by co-immunoprecipitation, by co-localization after flotation in density gradients and by confocal microscopy analysis of PrP^c^ and caveolin-1 distributions in a neuronal cell line (GN11) expressing caveolin-1 at high levels.

**Conclusions:**

We observed that, after antibody-mediated cross-linking or copper treatment, PrP^c^ was internalized probably into caveolae. We propose that following translocation from rafts to caveolae or caveolae-like domains, ^sec^PrP could interact with caveolin-1 and induce signal transduction events.

## Background

The tremendous advances in the comprehension of signal transduction mechanisms have been based mostly on the use of cultured cells and we know quite a lot of informations about apoptosis regulation, cell survival and cell fate. Signal transduction in neurons is used mainly to trigger cell survival and differentiation , but much less is known about the constituents participating to the transduction cascade especially as far as protein kinase family members acting downstream are concerned [1].

MAP kinase (ERK1/2) has been intensively studied in neurons because of its participation to hippocampal mechanisms leading to learning and memory consolidation [2]. How this kinase is recruited by signalosomes is a matter of controversy, but studies carried out by Lisanti and coworkers point to caveolin 1 and the caveolar-raft system as possible recruitment sites. However, this point has not been further investigated ,while an inverse relationship between ERK 1/2 and caveolin 1 cellular levels, has been clearly detected [3,4].

Fyn kinase, a member of src family kinase, unlike ERK1/2 has clearly been shown to be recruited in membrane microdomains and to interact there with ephrin A. Davy et al interestingly proposed that a transmembrane adaptor may be involved in coupling ephrin A activation to signal transduction Fyn kinase-mediated [5].

## Results and discussion

It is not understood to which extent these data can be applied to nerve cells. We have examined the role played by membrane microdomains in signal transduction generation using a hypothalamic neuronal cell line (GN11) where caveolin 1 gene is expressed at high levels. Since it has been reported that in neurons the cellular prion protein participates to signal transduction by activating Fyn kinase [6], GN11 cell have been transfected with a novel PrP^c^ construct allowing to reach an high efficiency-transfection procedure, in order to compare ERK1/2 and Fyn kinase activity in normal vs transfected cells. Moreover, we adopted a previously described procedure to activate PrP^c^ in membrane microdomains.

The results indicate that signal transduction activation by clustering PrP^c^ in caveolae, triggers a de-phosphorylation of ERK1/2 and a phosphorylation of Fyn kinase which became a caveolar constituent as judged from confocal microscopy evidences.

Studies regarding the functional significance of caveolae or caveolae-like structures in neuronal cells are difficult because most of neural cell lines available do not express or express at very low level caveolin 1 gene thus impairing caveolae formation [7]. For example, several neuroblastoma cell lines which are prone to transfection by PrP^c^ gene constructs, are difficult to differentiate and for this and other reasons do not express caveolin 1 gene [8]. Some years ago by chance we contacted a group using a line formed by immortalized hipothalamic neurons (GN11) rapidly proliferating and thus prone to transfection procedures, which on the other hand rapidly differentiated after treatment with TPA [9]. In differentiated cells caveolae were particularly abundant: in cells transfected with a PrP gene construct by immunoprecipitation using the 3F4 anti PrP^c^ antibody it has been possible to separate by western blot a series of bands ranging from 27 to 42 kD corresponding to the described various forms of PrP^c^ at different extent of glycosylation. These experiments carried out in cells exposed to 35 S methionine detecting radioactivity by conventional autoradiography, confirm previous results indicating that the formation of caveolae by TPA-induced differentiation is paralleled by an increased formation of prion protein and probably by its co-localization with caveolin 1 as suggested by the presence in the immunoprecipitate of a doublet corresponding to the mobility of caveolin 1. Sequencing will permit to unequivocally confirm these assumptions.

Lisanti and coworkers [7] have carried out intensive studies regarding transduction of signals occurring when caveolar complexes are activated. Caveolin 1 probably after phosphorylation, seems to be able to influence MAP kinase p42/44 (ERK1/2) activity. It is not clear if this typically cytosolic kinase is recruited by the caveolar complex or if its activity is modulated at the level of phosphorylation (MAP kinase kinase or MAP kinase phosphatase). In GN11 cells we detected high levels of constitutive ERK1/2, which did not vary in the different conditions under which GN11 cells were grown.

What appears to change dramatically is the phosphorylation status and the most de-phosphorylating (inactivating) procedures are PrPc cross linking induced by antibodies especially in the presence of copper. These data confirm the importance of an internalization process induced by cross linking and leading to a strong binding of PrP^c^ to caveolin 1 previously described. The results reported here suggest that following the formation of the caveolar complex, protein phosphatases, recently detected in membrane microdomains may be recruited and activated.

Several studies have focused on the role of copper ions in the physiology of the prion protein which is seen as a copper transporter [10,11]. Our data point more to a role of this ion in the binding to N-terminal octarepeats and in maintaining a PrP^c^ conformation capable of interacting functionally with other proteins as caveolin 1. For example, copper is unable to influence MAP kinase phosphorylation status.

It has been shown that one member of src family kinases (Fyn kinase) identified in rafts, participates to signal transduction events triggered by PrP^c^ inside caveolae of differentiated neuroectodermal cells, the evidence was obtained through co-immunoprecipitation studies. We have first identified the phosphorylated form in GN11 cells then we demonstrated that PrP^c^, caveolin 1 and Fyn kinase co-localize within caveolae using a novel technique developed by Santi and coworkers. It is shown that Fyn kinase is expressed at high levels in GN11 cells and that the levels do not change after cell treatment with copper ions or TPA or both. Even cross-linking using specific antibodies has any effect, however when anti P-tyrosine antibodies were employed it was clear that the P- form of Fyn kinase undetectable in control cells is rapidly augmented in PrP^c^-transfected cells. Moreover the phosphorylated form is favored (stabilized) when cells were previously exposed to copper ions. Thus in the same conditions under which ERK-1/2 is de-phosphorylated, the levels of P-Fyn increase dramatically. Results reported moreover indicate that cav1, PrP^c^ and Fyn kinase appear to share the same cellular sites: in caveolae first, then in caveosomes when cells are incubated for at least 1 hr at 37° C. This pattern is in accordance with a similar pattern observed using anti-PrP and anti cav1 antibodies.

## Conclusions

Multiproteic caveolar complexes represent a sophisticated membrane organization involved in signal transduction. Their efficiency is linked to the insertion of proteins in a restricted membrane area (50-100 nm) where the generation of a signal has a vectorial and oriented characteristic and allow the recruitment also of low abundance proteins (as for example PrP^c^) in order to generate signalling pathways which in neural cells may control differentiation and cell survival [12-15]. Results described here and previously indicate that downstream signalling is connected not only to modification of kinases as Fyn, but also to de-activation of MAP kinase which, on the basis of published evidence, is connected to the regulation of cell proliferation [16]. Whether MAP and Fyn kinases interact within caveolae and what kind of substrates undergo phosphate turnover, are the objective of ongoing experiments.

PrP^c^ in neurons has been reported to be involved in synaptic transmission and in hippocampal neurons to participate to long term memory retention [17,18]. Its role in organizing membrane microdomains multiprotein complexes as shown recently [19] is consistent with these functions.

An exciting recent result reported by Ghoshal et al.[20] detected a co-distribution of amyloid β plaques and spongiform degeneration in familial Creutzfeldt-Jakob disease with the E200K-129M haplotype. It is likely that both β amyloid and PrP^sc^ formation occur in rafts and therefore any variation in PrP^c^ may lead to β amyloid aggregation and spongiform degeneration.

## Methods

Methods are described in Toni M et al [21]

### Cell culture and transfection

Murine GN11 cells were grown at 37°C in 5% CO_2_ atmosphere in high glucose-culture medium (DMEM, Sigma, USA), supplemented with 10% heat-inactivated fetal calf serum (FCS, Cambrex BioWhittaker, USA), 2mM L-glutamine, penicillin (100 U/ml), and streptomycin (100 μg/ml).

Cells were transiently transfected by using Lipofectamine 2000 reagent (Invitrogen, USA), following manufacturer's instructions. For cell differentiation, phorbol ester 12-O-tetradecanoylphorbol-13-acetate (TPA) (20 nM, Sigma, USA) was added to cells soon after the transfection step, depending on the experiment. For Western blot experiments, cells were trypsinized 24 hours after transfection and reseeded in 25 cm^2^ flasks to obtain the same number of transfected cells in each flask. To assess the Erk 1/2 phosphorylation status, cells were serum-starved for 4 hours before antibody-mediated ligation of PrPc. Depending on the experiments, 30 μM PP2 (Calbiochem, USA) was added to the medium for 4 hours. In both immunoblotting and immunocytochemistry experiments, 48 hours after transfection, antibody-mediated stimulation was carried out first by incubating (10, 20, or 30 min, 37°C) intact live cells withMab 3F4, Mab Dpl 79, or anti-?-actin Pab and then with secondary anti-mouse or anti-rabbit antibodies for additional 10–120 min. (37°C), depending on the experiment. Then, cells were lyzed in lysis buffer (50mM Tris-HCL, pH 7.5, 2 mM EDTA, 100 mM NaCl, 1% Triton X100, 5 mM NaF, 1 mM Na3VO4, 10 mM β-glycerolphosphate, and proteases inhibitors) or fixed with 4% paraformaldehyde, for western blot or immunofluorescence analysis, respectively.

### Coimmunoprecipitation assay

In Cav-1 immunoprecipitation experiments, cells transiently expressing the HA-tagged PrPc (one confluent 25 cm^2^ flask for each sample) were lyzed in 500 μl of a buffer containing 10mM Tris (pH 7.5), 150mM NaCl, 1mM EDTA, 1% Triton X-100, 0.5% NP40, 10% glycerol, and protease inhibitors. Precleared supernatants were immunoprecipitated using anti-Cav-l Pab (3 μg/ml), followed by incubation with protein A Sepharose. After washings, immunoprecipitated samples were processed by western blot to detect PrPc. For sample deglycosylation, immunoprecipitates were treated (24 hours, 37°C) with PNGase-F (5 U) (Roche Molecular Biochemicals, Germany) . For PrP immunoprecipitation, the same protocol described above was followed, except for the use of an anti-HA tag antibody to immunoprecipitate. The presence of Cav-1 in the immunoprecipitated samples was then assayed by immunoblotting with anti Cav-1 Pab.

### GST-binding assay

Recombinant full-length forms ofmorPrP (23–231) and hur- Dpl (28–152) were generated in, and purified from, *Escherichia coli*. To provide the epitope specific for Mab 3F4, morPrP carried two Mets at positions 108 and 111 (L108M, V111M). Murine Cav-1 forms were recombinantly obtained as GST-fusion proteins. For binding assays, 0.2 μM of Glutathione STransferase (GST) or GST-Cav fusion proteins (prebound to glutathione-Sepharose beads) were incubated overnight with equimolar amounts of morPrP or hurDpl (in a final volume of 0.25 ml), under continuous shaking at 4°C. Proteins bound to glutathione-Sepharose beads were eluted, washed, and immunoblotted to detect the presence of PrP or Dpl. In competition experiments, equimolar amounts of either Ab · Tg or C-20 antibodies were present during the entire incubation period.

## Competing interest

The author declare that they have no competing interests.
